# Nursing and its Essential Role in the Vaccination against COVID-19: New Challenge in a Pandemic Scenario

**DOI:** 10.17533/udea.iee.v39n3e01

**Published:** 2021-11-05

**Authors:** R. Mauricio Barría P

**Affiliations:** 1 RN, MSc, DrPH. Director of the Institute of Nursing, Faculty of Medicine, Universidad Austral de Chile. email: rbarria@uach.cl Universidad Austral de Chile Faculty of Medicine Universidad Austral de Chile Chile rbarria@uach.cl

**Keywords:** SARS-CoV-2, COVID-19, mass vaccination, vaccination refusal, nursing.

To the date of publication of this editorial, we have gone through over 20 months of facing the complex and challenging SARS-CoV-2 pandemic since the first case was reported in late 2019. The consequences globally have been significant not only due to the unprecedented morbidity and mortality throughout the world, but also as effect of the drastic changes taking place in the usual dynamics in the individual, family, and collective settings, given the overall interruption of habitual functions and operations in the distinct contexts of daily life, sustaining only those activities considered essential.

Since then, varying non-pharmacological interventions and public health measures have been implemented aimed at stopping or slowing down the propagation of the virus during the first stages of the pandemic, including some controversial and others that have proven effective, like obligatory use of face masks, physical distancing and restrictions on mobility due to the establishment of quarantines and home confinement, travel restrictions, frontier closings, and intensive tests of trace and follow-up to contacts.(1)

Nevertheless, and as it has occurred with other infectious diseases, in spite of the measures described, requirement of a vaccine was seen as an urgent intervention to confront the pandemic advance. As has been the experience during other moments, the vaccination has contributed in large degree to global health being considered one of the most important accomplishments of science and medicine, given that together with mitigating the spread of infectious diseases, it reduces the economic impact in health systems.(2) Consequently, the rapid dissemination of the SARS-CoV-2 coronavirus obligated different laboratories throughout the world to develop vaccines at a fast race upon isolating the virus for the first time and sequencing its genome in early 2020. Efforts to develop a vaccine today account for studies in over 100 products, several of which have already been authorized by regulatory agencies.(3)

In this perspective based on the good results shown by some vaccines, it is that, as of early 2021, different countries, through their governments, began to negotiate and acquire these products to implement mass vaccination programs against COVID-19 to contain greater propagation of SARS-CoV-2 and, thus, allow return to the habitual activities of the community and society in general. However, the capacity of the vaccination program to control the disease, as with other vaccination programs, like that developed to confront influenza, will depends on its reach in the community and, in the case of the COVID-19 vaccine, it will be required to capture at least 80% of the population during the first year of vaccination.(4)

It is within this panorama that nurses globally have been fundamental in this new task, adding to the contribution they have already made not only by their incessant work in the front line, participating in the health teams that provide care to critical patients with COVID-19, but at different levels participating in information campaigns and control tasks to verify compliance with preventive contagion measures. From their positioning and recognition in the community and taking advantage of the direct contact they have daily with users and patients in the different settings in which they work, nursing professionals participate by educating the vaccine receptors to raise public awareness besides participating directly in their administration, a great task to achieve broad coverage in a short time. As has occurred with other vaccination plans and programs throughout the life cycle, primary and community care nurses are in charge of guaranteeing the manipulation, storage, and safe administration of the vaccines and have contributed to promoting the vaccination, helping to design and carry out efficient campaigns. Due to this, today they constitute a crucial factor for the success of mass vaccination programs against COVID-19.

What today is relatively clear is that, considering the satisfactory efficacy reported for different vaccines (> 80%) and, additionally, knowing that the protection is short, immunization is required of a large proportion of the population so that with this coverage there is some possibility of obtaining herd immunity to block continuous transmission of SARS-CoV-2.(4) Uncertainty is greater, given that it is recognized that the first-generation vaccines are effective to reduce serious disease due to SARS-CoV-2 and deaths due to COVID-19, but their full effectiveness has not been assured against emerging variants.

The new challenge in this pandemic scenario is to face the need to promote trust and acceptance of the vaccines within a context of misinformation and mistrust, given that - as a consequence of the speed of the investigation and production of new vaccines, many people are not only skeptical about their safety if not totally resistant to the idea of getting vaccinated. Due to this, beyond the complex task of implementing vaccine administration plans in different places, doubts about vaccines should be addressed and promote trust in them(5) because sufficient evidence exists that the success of a vaccination program depends on its coverage to achieve herd immunity, but it is equally clear that indecision or uncertainty regarding the vaccines has the potential to undermine such programs.(6,7) It must be highlighted that the COVID-19 vaccine continues being the most effective medium to achieve control of the pandemic and, likewise, people not vaccinated continue being at substantial risk of infection, serious disease, and death, especially in areas with high levels of SARS-CoV-2 community transmission.(8)

Given the indecision around vaccines, the creation of herd immunity through vaccination is likely to be a challenge in many countries and, consequently, communication and the resulting public awareness is likely the most crucial role played currently by nurses in the vaccination process. For such, primary and community care nurses must help in the promotion and acceptance of the vaccines through evidence-based interventions and assume a fundamental role in allowing adequate information to be transmitted and promoted in the adequate moment, in the adequate level, and in the adequate format.(5,6) In parallel and given the experience of the front-line nurses during the pandemic, they can communicate how the vaccine can mitigate the consequences of COVID-19 and, additionally, be an example for the population by getting vaccinated and to be able to model the behavior that others should adopt in the face of the possibility of contagion.

The COVID-19 pandemic has evidenced the need to demonstrate the timely, creative, and innovative response capacity of health staff and prominently nurses in clinical care and community contexts to face an international disaster that, until now, has lasted for almost two years and which has required different strategies, interventions, and perspectives. Nursing participation during this critical period has been expressed in the essential function in their clinical settings when providing patient care, reorganizing routines, spaces, and work teams to respond to the high demand. In other scenarios, they have supported the public health initiatives when participating in prevention and promotion actions, in screening for the disease, in the follow up of contacts, and in the control and surveillance of measures adopted by central and local governments, among other actions. But, currently, a responsibility and greater challenge is their leading role in mass vaccination plans against COVID-19 in which besides providing security in vaccine management processes regarding storage, distribution, and administration they must provide the necessary trust based on their credibility and position in the community, and employing the necessary communication tools to permit appropriate vaccination coverage that provides the required protection to the population.
